# Characteristics of peripheral white blood cells in COVID-19 patients revealed by a retrospective cohort study

**DOI:** 10.1186/s12879-021-06899-7

**Published:** 2021-12-09

**Authors:** Xunliang Tong, Anqi Cheng, Xueting Yuan, Xuefeng Zhong, He Wang, Wei Zhou, Xiaomao Xu, Yanming Li

**Affiliations:** 1 Department of Pulmonary and Critical Care Medicine, Beijing Hospital, National Center of Gerontology; Institute of Geriatric Medicine, Chinese Academy of Medical Sciences, Beijing, People’s Republic of China; 2 Tobacco Medicine and Tobacco Cessation Center, Center of Respiratory Medicine, China-Japan Friendship Hospital, Beijing, China; 3 WHO Collaborating Center for Tobacco Cessation and Respiratory Diseases Prevention, Beijing, China; 4 National Clinical Research Center for Respiratory Diseases, Beijing, China; 5 Institute of Respiratory Medicine, Chinese Academy of Medical Sciences, Beijing, People’s Republic of China; 6 The Key Laboratory of Geriatrics, Beijing Institute of Geriatrics, Beijing Hospital, National Center of Gerontology, National Health Commission, Institute of Geriatric Medicine, Chinese Academy of Medical Sciences, Beijing, People’s Republic of China

**Keywords:** COVID-19, Lymphocytes, Eosinophils, Prognosis, Immune response

## Abstract

**Background:**

Peripheral hematological changes in severe COVID-19 patients may reflect the immune response during SARS-CoV-2 infection. Characteristics of peripheral white blood cells as early signals were needed to be investigated for clarifying its associations with the fatal outcomes in COVID-19 patients.

**Methods:**

A retrospective cohort study was performed and the hospitalized COVID-19 patients were recruited in wards of Sino-French New City Branch of Tongji Hospital in Wuhan, Hubei province, China. Characteristics of peripheral white blood cells in survivors and non-survivors were analyzed. Comparison among patients with different level of eosinophils was performed.

**Results:**

Of 198 patients included in this study, 185 were discharged and 13 died. Levels of eosinophils, lymphocytes and basophils in non-survivors were significantly lower than those in survivors. Death rate in low eosinophils group was higher and no patient died in normal eosinophils group (16.7% vs 0, P < 0.001). The proportion of patients in low eosinophils group who used glucocorticoids was higher than in normal eosinophils group, but glucocorticoids usage was not an indicator for death in subgroup analysis in low eosinophils patients. Moreover, positive correlation was found between the counts of lymphocytes and eosinophils in patients with glucocorticoids use but not in patients without the treatment.

**Conclusions:**

Hematological changes differed between survivors and non-survivors with COVID-19. Lymphopenia and eosinopenia could be predictors for poor prognosis of COVID-19 patients. Initial counts of eosinophils may guide us in usage of glucocorticoids for COVID-19 treatment.

**Supplementary Information:**

The online version contains supplementary material available at 10.1186/s12879-021-06899-7.

## Background

The outbreak of COVID-19 caused by severe acute respiratory syndrome coronavirus 2 (SARS-CoV-2) has rapidly spread throughout the world [1, 2]. The clinical manifestation of COVID-19 could specifically display in a wide spectrum, which is so far mostly mild and self-limiting. Besides, other COVID-19 patients show severe viral pneumonia with respiratory dysfunction, even including several organs failure, resulting in a 2% to 3% mortality rate worldwide [3].

With advanced knowledge, immune system dysfunction triggered by SARS-CoV-2 was observed in COVID-19 patients. In mild cases, immune responses were efficiently established to curb the viral replication, while in severe cases, uncontrolled inflammation and the microcirculation dysfunctions together lead to viral sepsis with immunologic impairment [4]. The severity of disease was associated with immunological impairment. Especially, in some life-threaten cases, SARS-CoV-2 could trigger catastrophic damage to the human immune system resulting in death at their worst.

Unfortunately, our understanding of immune response to SARS-CoV-2 is extremely limited until now. Many scholars speculated that the interaction of SARS-CoV-2 and host could be referring to the other coronavirus because of the highly similarity in the sequence homology in coronavirus family [5]. Previous study mainly focused on the immune dysfunction caused by severe acute respiratory syndrome coronavirus (SARS-CoV) and Middle East respiratory syndrome coronavirus (MERS-CoV), respectively. Coronavirus infections (SARS and MERS) are confirmed to activate both innate and adaptive immune responses [6, 7]. In simply it means that the changes of peripheral blood cells could reflect the immune damage caused by virus infection.

Lymphocytes play a crucial role in maintaining immune homeostasis during virus infection, especially SARS-CoV-2 [8]. Several cohort studies have reported that lymphopenia can predict prognosis in COVID-19 patients [9, 10]. In addition, a few studies found that the eosinopenia was also associated with poor prognostic features [11, 12]. Thus, the differentiation of peripheral white blood cells may indicate the immunologic impairment at the early stage of the disease. However, the risk factors for the changes of peripheral blood cells, especially eosinophils (EOS) in the prognosis have not been well addressed yet. This retrospective cohort study was performed to assess the value of peripheral white blood cells in COVID-19 patients.

## Methods

### Study design

Patients were diagnosed of COVID-19 according to World Health Organization interim guidance. COVID-19 positivity was confirmed of nucleonic acid for SARS-CoV-2 by throat swab. As one-time supportive task led by China National Health Commission, patients who were hospitalized at designated wards in the Sino-French New City Branch of Tongji Hospital in Wuhan, Hubei province, China were under treatment by medical team from Beijing Hospital. This retrospective cohort study was implemented from Feb 8th to March 15th, 2020 and all COVID-19 patients were consecutively recruited. We excluded patients with hematological disease or a blood transfusion in a week after admission. The severity of the disease was assessed according to the Seventh Version of the Novel Coronavirus Pneumonia Diagnosis and Treatment Guidance from the National Health Commission of China. CURB-65 score were also calculated and patients were divided into 3 groups: low risk (0–1 point), intermediate risk (2 points) and high risk (3–5 points) [13].

The study was approved by Ethics Committee of Beijing Hospital (2020BJYYEC-046-01).

### Medical data extraction

The clinical data, including demographics information; clinical symptoms and signs; underlying diseases; laboratory results; the most intense level of oxygen support; treatment and clinical outcomes, were extracted from electronic medical records. The whole laboratory evaluation consisted complete blood cell counts, biochemical and coagulation indices and so forth. The differential peripheral blood indices were detailed recorded. Laboratory data at baseline were recorded in the first 24 h after admission According to the level of circulating EOS counts on admission, COVID-19 patients were divided into two groups: low EOS group (< 0.02 × 10^9^/L) and normal EOS group (≥ 0.02 × 10^9^/L). The end point was written of discharging from hospital or death. The differences in clinical characteristics and laboratory findings in patients with different outcome would be addressed. Longitudinal tracing of laboratory indices during the hospitalization was performed and the endpoint laboratory examinations were performed. All the data were entered into a computerized database and checked by two experienced physicians independently.

### Statistical analysis

Continuous variables were described using median and interquartile range (IQR). Categorical variables were described as number (%). Non-normal distributed continuous data were compared using Mann–Whitney–Wilcoxon test. Categorical data were compared using *X*^2^ test or the Fisher exact test. Correlations between variables were analyzed using the Spearman's rank correlation. Correlation strength was selected by an absolute correlation |*r|*> 0.2 and the selected correlation were plotted as an undirected network graph. All tests were 2-sides, and a *P* value < 0.05 was considered statistically significant. Data was analyzed using IBM SPSS Statistics software (version 19.0).

## Results

### Demographic information and laboratory findings at baseline of survivors and non-survivors

A total of 198 patients confirmed severe COVID-19 were enrolled in this study. According to the clarified outcome (discharged or deceased), patients were divided in two groups: survivors and non-survivors. The median age of patients and the gender distribution between two groups (survivor group and non-survivor group) was basically the same. Majority of the included patients in both two groups were with comorbidity and more than half of the patients had at least one underlying disease. The ranking of the underlying disease was hypertension (40.0%), diabetes (16.7%), chronic respiratory diseases (5.7%), cardiovascular diseases (5.2%) and so on. Among the underlying disease, the percentage of malignant disease of patients in the non-survivor group is higher than it in the other group. But these patients with malignancy were all in stable stage and hadn’t received any relevant surgical or chemotherapy treatment within three months. The commonest symptoms on admission were fever and cough, followed by fatigue and sputum production in both two groups. All these information was listed in Table 1.

**Table 1:** Demographic and laboratory findings at baseline of COVID-19 hospitalized patients

Characteristics	Total (n = 198)	Survivor (n = 185)	Non-survivor (n = 13)	*P* value
Demographic				
Age, median (IQR), years	63(48,69)	62(48,69)	68(58,84)	0.065
Gender, Male, n (%)	99(50.0%)	90(48.6%)	9(69.2%)	0.251
Smoking, n (%)	6(3.1%)	6(3.3%)	0	1.000
Comorbidities, n (%)				
Chronic respiratory disease	11(5.7%)	11(6.1%)	0	1.000
Malignancy	7(3.6%)	4(2.2%)	3(23.1%)	**0.007**
Hypertension	76(40.0%)	72(40.7%)	4(30.8%)	0.568
Diabetes	32(16.7%)	30(16.8%)	2(15.4%)	1.000
Cardiovascular disease	10(5.2%)	9(5.0%)	1(7.7%)	0.513
Chronic kidney disease	6(3.1%)	6(3.4%)	0	1.000
Signs and symptoms, n (%)				
Fever	153(79.7%)	142(79.3%)	11(84.6%)	1.000
Chills/shivers	42(21.2%)	40(21.6%)	2(15.4%)	0.739
Cough	124(64.9%)	117(65.7%)	7(53.8%)	0.386
Productive cough	61(31.9%)	57(32.0%)	4(30.8%)	1.000
Chest pain/chest congestion	38(19.9%)	35(19.7%)	3(23.1%)	0.725
Dyspnea	66(34.6%)	61(34.3%)	5(38.5%)	0.759
Diarrhea	57(29.7%)	54(30.2%)	3(23.1%)	0.758
Fatigue or myalgia	83(41.9%)	77(41.6%)	6(46.2%)	0.749
CURB-65				** < 0.001**
0–1	170(86.7%)	170(92.9%)	0	
2	14(7.1%)	13(7.1%)	1(7.7%)	
3–5	12(6.1%)	0(0)	12(92.3%)	
Laboratory findings				
Hematologic				
White blood cells, × 10^9^/L	5.46(4.19,7.10)	5.42(4.17,6.83)	6.73(3.93,9.37)	0.248
Neutrophils, × 10^9^/L	3.43(2.43,4.81)	3.36(2.40,4.66)	5.58(2.99,8.41)	**0.024**
Neutrophil percentage, %	66.5(55.6,74.0)	65.5(55.4,73.1)	83.6(76.4,89.8)	** < 0.001**
Lymphocytes, × 10^9^/L	1.02(0.78,1.52)	1.08(0.78,1.57)	0.50(0.33,0.82)	** < 0.001**
Lymphocyte percentage, %	23.4(15.1,31.6)	24.5(16.0,32.8)	10.9(5.25,16.9)	** < 0.001**
Lymphocytes < 0.8 × 10^9^/L, n (%)	75(37.9%)	65(35.1%)	10(76.9%)	**0.005**
Neutrophil-to-lymphocyte ratio	3.15(1.80,5.59)	3.05(1.76,5.17)	8.43(4.52,18.61)	** < 0.001**
Monocytes, × 10^9^/L	0.46(0.33,0.62)	0.47(0.34,0.63)	0.33(0.21,0.60)	0.168
Monocyte percentage, %	8.5(6.5,10.3)	8.7(6.9,10.4)	5.9(3.5,8.2)	** < 0.001**
Eosinophils, × 10^9^/L	0.03(0.00,0.09)	0.03(0.00,0.10)	0.00(0.00,0.00)	** < 0.001**
Eosinophil percentage, %	0.55(0.00,1.70)	0.70(0.00,1.75)	0.00(0.00,0.00)	** < 0.001**
Eosinophil < 0.02 × 10^9^/L, n (%)	78(39.4%)	65(35.1%)	13(100.0%)	** < 0.001**
Basophils, × 10^9^/L	0.01(0.01,0.02)	0.01(0.01,0.02)	0.00(0.00,0.01)	**0.001**
Basophil percentage, %	0.2(0.1,0.4)	0.2(0.1,0.4)	0.0(0.0,0.0)	** < 0.001**
Red blood cells, × 10^12^/L	4.10(3.70,4.45)	4.10(3.75,4.46)	3.39(1.77,4.37)	**0.013**
Platelets, × 10^9^/L	225(163,301)	232(171,305)	145(76,241)	**0.007**
PLT < 100 × 10^9^/ L, n (%)	12(6.1%)	8(4.3%)	4(30.8%)	**0.004**
Hemoglobin, g/L	124(114,137)	125(115,137)	107(55,136)	**0.021**
Biochemical test				
Albumin, g/L	36(32,39)	36(32,39)	33(29,36)	**0.023**
ALT, U/L	22(15,39)	23(15,40)	19(17,33)	0.482
AST, U/L	27(19,38)	27(19,37)	47(36,58)	**0.005**
Creatinine, μmol/L	70(58,86)	69(58,83)	99(61,114)	**0.019**
LDH, U/L	261(206,328)	258(205,318)	400(302,674)	**0.001**
Other indices				
Erythrocyte sedimentation rate, mm/h	40(18,64)	39(18,64)	50(12,71)	0.953
Serum ferritin, ng/mL	522(310,893)	480(308,799)	1968(1520,3507)	**0.001**
IL-6, pg/mL	11.6(4.2,27.1)	9.3(4.0,21.1)	41.6(23.2,65.3)	**0.003**
C-reactive protein, mg/L	23.1(3.2,50.2)	16.8(2.8,45.0)	72.4(44.8,165.0)	** < 0.001**
Troponin↑, n (%)	24(18.0%)	14(11.7%)	10(76.9%)	** < 0.001**
NT-proBNP, pg/mL	157(64,411)	127(62,335)	554(483,995)	** < 0.001**
PT, s	13.8(13.3,14.3)	13.7(13.2,14.2)	14.9(13.9,16.0)	**0.002**
APTT, s	39.7(36.6,43.9)	39.6(36.6,43.4)	45.4(37.8,47.2)	0.105
FIB, g/L	4.71(3.73,5.85)	4.65(3.75,5.81)	4.87(2.61,6.11)	0.763
D-Dimer, μg/mL	0.91(0.46,1.80)	0.76(0.46,1.67)	2.01(1.10,5.10)	**0.009**
D-Dimer↑, n (%)	116(63.7%)	105(62.1%)	11(84.6%)	0.138

**Fig. 1:**
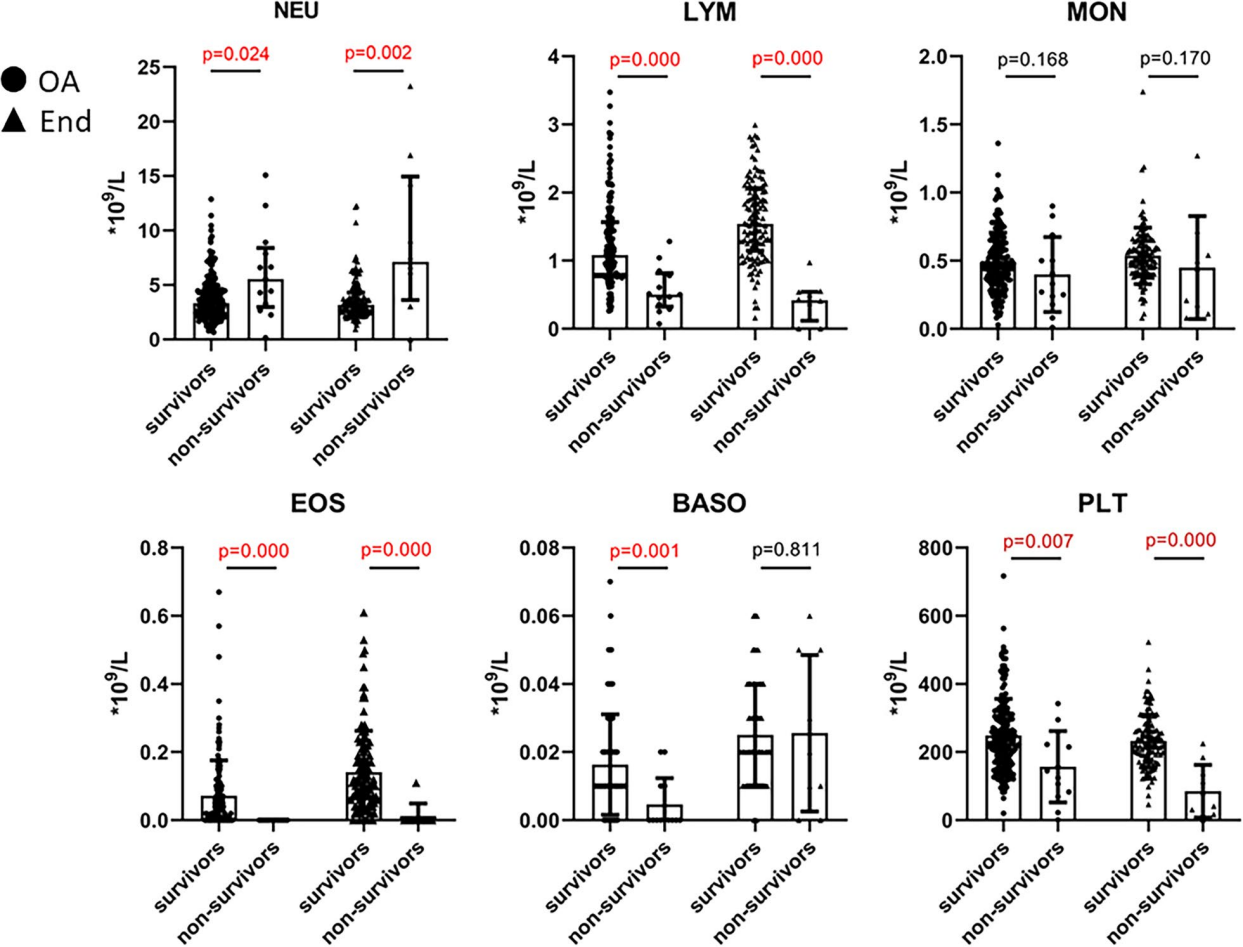
Characteristics of peripheral blood cells between survivors and non-survivors. Figure shows the counts of peripheral blood cells among survivors and non-survivors on admission (OA) and end hospitalization (End)

Hematologic profile on admission varied among patients between survivor and non-survivor groups. Compared to survivors, the counts of eosinophils (EOS) and basophils (BASO) in non-survivors were too low to be detected. Lymphocytes (LYM), monocytes (MONO) and platelets (PLT) were considerably lower in non-survivors compared with survivors. (LYM: 10.9% vs 24.5%, *P* < 0.05; MONO: 5.9% vs 8.7%, *P* < 0.05; PLT: 145[76, 241] × 10^9^/L vs 232[171, 305] × 10^9^/L, *P* < 0.05). Level of neutrophils (NEU) and the ratio of neutrophils-to-lymphocytes ratio (NLR) were significantly higher in non-survivors than survivors at baseline (NEU: 83.6% vs 65.5%, *P* < 0.05; NLR: 8.43 vs 3.05, *P* < 0.05). The percentage alteration was in consistent with the absolute counts change for each analyzed peripheral blood cells. Moreover, compared with survivors, levels of C-reactive protein (CRP), IL-6, and serum ferritin were significantly higher in non-survivors with statistical differences. Regarding the coagulation parameters, the prolonging of PT and increased levels of D-dimer were significantly higher in non-survivors compared to survivors (Table 1; Fig. 1).

### Treatment after enrollment and outcomes of COVID-19 patients

During hospitalization, the most intense level of oxygen support was recorded. Patients in survivor group were mostly under oxygen therapy by nasal cannula compared to patients in non-survivor group (47% vs 0, *P* < 0.05). The proportion of patients under invasive ventilation (IMV) in non-survivors was significantly higher than that in the other group (53.8% vs 2.2%, *P* < 0.05). More than 70% of the patients received antivirals, and Lopinavir/Ritonavir usage differed significantly between non-survivors and survivors (6.7% vs 100.0%, *P* < 0.05). According to the CURB-65 score, the proportion of patients with different grade showed significant differences between the two groups. Application of systematic glucocorticoids differed significantly between non-survivors and survivors (66.7% vs 22.5%, *P* = 0.002). At the end of the observing period, 185 (93.4%) patients were discharged and 13 (6.6%) patients died. (Table 2).

**Table 2:** Treatment after enrollment and outcomes of COVID-19 patients

	Total (n = 198)	Survivor (n = 185)	Non-survivor (n = 13)	*p* value
Oxygen therapy, n (%)				
Nasal cannula	87(43.9%)	87(47.0%)	0	** < 0.001**
Oxygen mask	4(2.0%)	3(1.6%)	1(7.7%)	0.257
NMV + High-flow nasal cannula	84(42.4%)	79(42.7%)	5(38.5%)	0.576
IMV	11(5.6%)	4(2.2%)	7(53.8%)	** < 0.001**
ECMO	2(1.0%)	2(1.1%)	0	1.000
Drugs, n (%)				
Oseltamivir	60(36.4%)	58(37.9%)	2(16.7%)	0.214
Arbidol	121(71.2%)	113(71.5%)	8(66.7%)	0.745
Lopinavir + Ritonavir	23(14.2%)	10(6.7%)	13(100.0%)	** < 0.001**
Ribavirin	9(5.6%)	8(5.4%)	1(8.3%)	0.672
Glucocorticoid	42(25.8%)	34(22.5%)	8(66.7%)	**0.002**
Hospital length of stay, days	14(10,18)	14(11,18)	8(4,12)	** < 0.001**
Time from illness onset to discharge, days	27(22,34)	27(23,34)	20(9,28)	**0.005**

### Effects on clinical characteristics of different EOS levels in COVID-19 patients

The age and gender distribution between low EOS group and normal EOS group showed no significant differences (age: 64 [53, 71] years vs 61 [47, 69] years, *P* = 0.126; gender (male): 52.6% vs 48.3%, *P* = 0.561). The body temperature on admission in the low EOS group was significantly higher than in normal EOS group (38.9 [38.4, 39.0] °C vs 38.5 [38.0, 39.0] °C, *P* = 0.011) and other symptoms did not differ significantly. The distributions of CURB-65 score of patients between the two groups were significantly different. The proportion of patients who used glucocorticoids in low EOS group was significantly higher than that in normal EOS group (44.8% vs 12.5%, *P* < 0.05). But in low EOS patients, the percentage of glucocorticoids usage in non-survivor group was not significantly different from that in survivor group (66.7% vs 40.0%, *P* = 0.117, Additional file 1: Table S1). In survivor group, high level of EOS and increasing levels of EOS was detected, whether with or without glucocorticoids usage (Additional file 1 Table S2). Duration of viral shedding in two groups showed no significant difference. Moreover, death rate in the low EOS group was significantly higher and no patient died in normal EOS group (16.7% vs 0, *P* < 0.05) (Table 3).

**Table 3:** Clinical characteristics of patients according to eosinophils level on admission

	EOS (< 0.02 × 10^9^/L) (n = 78)	EOS (≥ 0.02 × 10^9^/L) (n = 120)	P
EOS counts, median (IQR), × 10^9^/L	0.00(0.00,0.00)	0.08(0.04,0.15)	< 0.001
Age, median (IQR), yrs	64(53,71)	61(47,69)	0.126
Gender, male, n (%)	41(52.6%)	58(48.3%)	0.561
Signs and symptoms, n (%)			
Fever	64(85.3%)	89(76.1%)	0.120
Chills/shivers	18(23.1%)	24(20.0%)	0.605
Cough	46(61.3%)	78(67.2%)	0.403
Productive cough	61(31.9%)	22(29.3%)	0.535
Chest pain/chest congestion	16(21.3%)	22(19.0%)	0.689
Dyspnea	26(34.7%)	40(34.5%)	0.979
Diarrhea	17(22.7%)	40(34.2%)	0.088
Fatigue or myalgia	28(35.9%)	55(45.8%)	0.166
Highest temperature, °C	38.9(38.4,39.0)	38.5(38.0,39.0)	**0.012**
CURB-65, n (%)			** < 0.001**
0–1	59(76.6%)	111(93.3%)	
2	6(7.8%)	8(6.7%)	
3–5	12(15.6%)	0	
Glucocorticoid, n (%)	30(44.8%)	12(12.5%)	** < 0.001**
Duration of viral shedding, days	26(21,32)	26(20,33)	0.753
Hospital length of stay, days	15(10,18)	13(11,17)	0.609
Death, n (%)	13(16.7%)	0	** < 0.001**

### Changing features and correlation networks analysis for peripheral blood cells

**Fig. 2:**
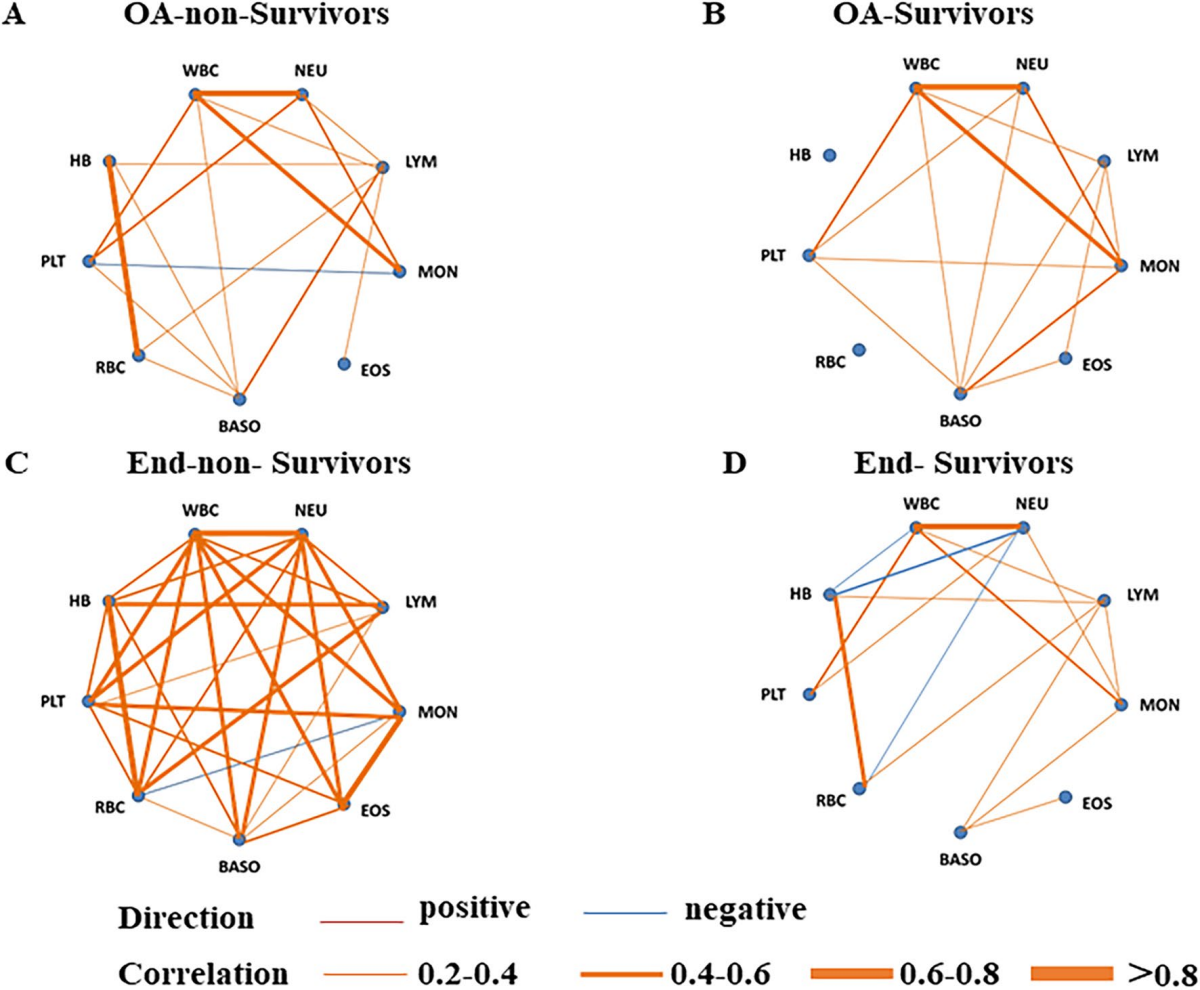
Correlation networks for peripheral blood cells among survivors and non-survivors. Networks showed different profiles of correlations in non-survivors (**A** and **C**) and survivors (**B** and **D**), on admission (**A** and **B**) and end hospitalization (**C** and **D**). The width of the edge showing stronger or weaker interactions is proportional to the absolute value of cell–cell correlation (|r|). Edges were shown only when |r|> 0.2. An orange edge indicates a positive correlation, and a blue edge indicates a negative correlation

A comparison of the levels of white blood cells at both baseline and endpoint was performed. Changes of EOS, LYM and NEU levels were different between groups. From baseline to endpoint, EOS and LYM count considerably increased in survivor group, while EOS and LYM count changed little in non-survivor group. In contrast, almost unchanged level of NEU was shown in survivors. All the information above was shown in Additional file 1: Table S3. We also observed a positive correlation between the counts of NEU and MONO (r = 0.549), NEU and PLT (r = 0.530) in non-survivors on admission. Before discharge, strong correlations between counts of NEU and MONO (r = 0.771), NEU and EOS (r = 0.735), NEU and BASO (r = 0.623) were observed in non-survivors. Furthermore, non-survivors showed similar positive correlations between the counts of EOS and BASO (r = 0.284) (Fig. 2).

**Fig. 3:**
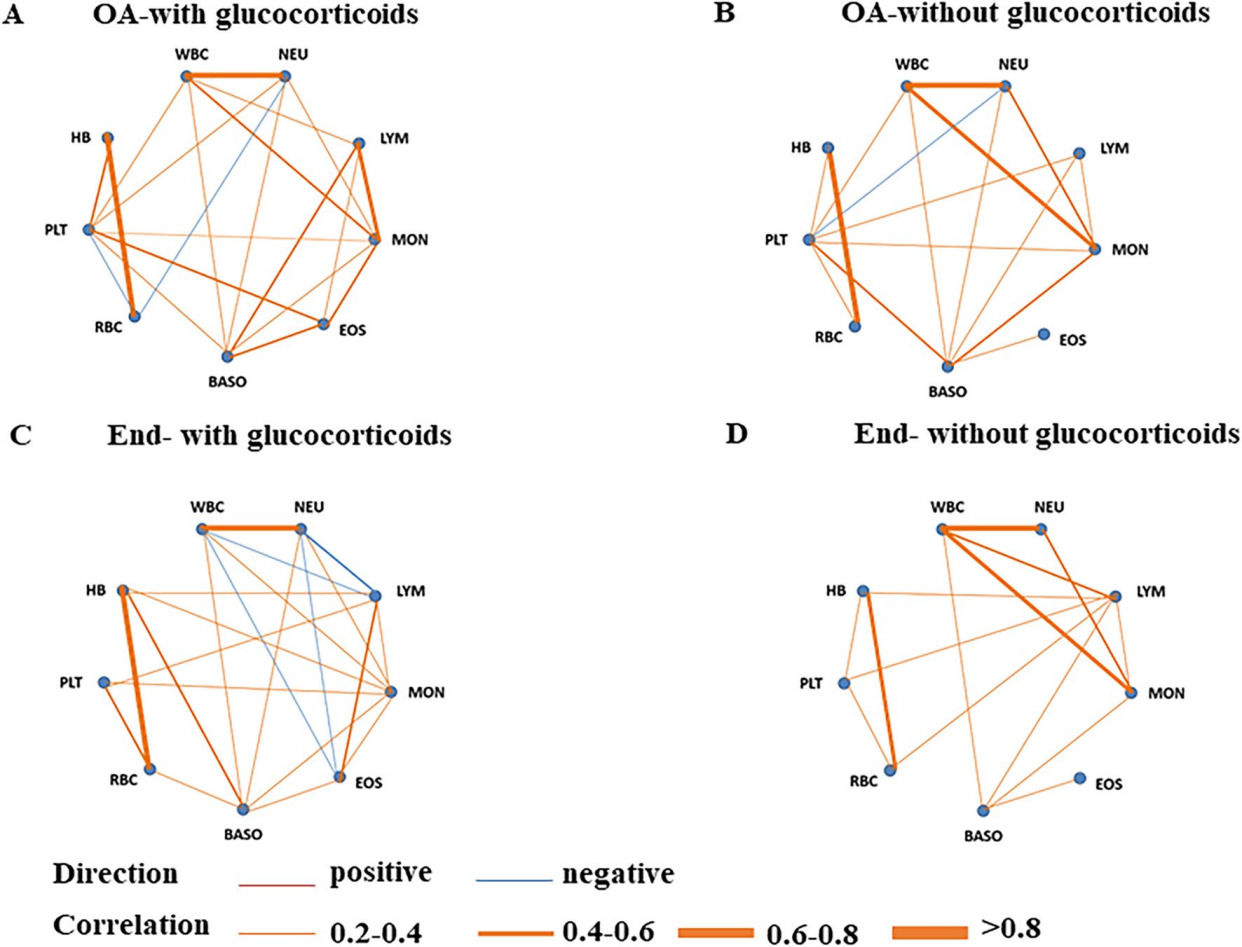
Correlation networks for peripheral blood cells among patients with and without glucocorticoids. Networks showed different profiles of correlations in patients with glucocorticoids (**A** and **C**) and without glucocorticoids (**B** and **D**), on admission (**A** and **B**) and end hospitalization (**C** and **D**). The width of the edge showing stronger or weaker interactions is proportional to the absolute value of cell–cell correlation (|r|). Edges were shown only when |r|> 0.2. An orange edge indicates a positive correlation, and a blue edge indicates a negative correlation

Patients after glucocorticoids therapy showed a negative correlation between the counts of WBC and LYM (r = − 0.265), but a positive correlation between WBC and LYM was observed in patients without glucocorticoids therapy (r = 0.531). After glucocorticoids treatment, the counts of EOS negatively correlated with NEU (r = − 0.288), but no correlation was observed before the treatment (r = 0.058). A similar correlation was observed between the counts of LYM and EOS in patients received glucocorticoids therapy (r = 0.454), but no correlation was found without the treatment (r = 0.020) (Fig. 3).

## Discussion

As the classification of survivors and non-survivors was observed in this retrospective cohort study, the differential features of peripheral white blood cells were analyzed. Previous study demonstrated that severe patients tend to have lower lymphocytes counts, higher leukocytes counts and neutrophil–lymphocyte-ratio (NLR), as well as lymphopenia has been reported as a predictor of prognosis in COVID-19 patients [9, 14, 15]. Our data also revealed that the initial counts of lymphocytes, eosinophils, and basophils of COVID-19 patients were much lower in non-survivors compared with the counts of above indices in survivors, which was consistent with conclusion of other studies [11, 12].

Eosinophils are linked to immune response conferring host protection against viruses and eosinopenia has been observed in different acute inflammation situation as pneumonia [16–18]. A recent report by Xie et al. proved that COVID-19 patients with low EOS counts were likely to have more severe symptoms such as fever, fatigue, shortness of breath, more lesions in chest CT, radiographic aggravation, longer length of hospital stay and course of disease [19]. Our study also indicated that patients with low EOS on admission showed as a predictor for in-hospital death. Eosinopenia may be the result of rapid sequestration of circulating eosinophils mediated by the overwhelming release of inflammatory cytokines, including thermogenic ones (such as IL-1, IL-6) [20]. In addition, effects of glucocorticoids on hematological and immunological indicators were significant, especially the decrease in counts of eosinophils.

Although our understanding of the specific innate and adaptive immune response to SARS-CoV-2 is relatively limited, the hematological changes may reflect a homeostatic mechanism to prevent systemic over-activation of inflammation. SARS-CoV-2 RNA and proteins interact with various pattern recognition receptors can initiate antiviral immune responses which characterized by differentiation and proliferation of various immune cells with immune mediator production and release, especially lymphocytopenia and elevated level of IL-1β, IFN-γ, IP-10 and IL-17, regulating viral replication and spreading within the host [21, 22]. SARS-CoV-2 has been proven to induce remodeling of peripheral lymphocytes, and a more robust humoral immune response occurs in severe infection [23]. The decreased production, apoptosis and redistribution of lymphocytes may together lead to circulating lymphopenia [24]. In addition, eosinophils are recruited from the blood circulation into the inflammatory focus, modulating immune responses through releasing a serious of cytokines and other mediators, as well as by a broad spectrum of immune mechanisms [25]. In short, uncontrolled SARS-CoV-2 infection and the immune response may cause a systemic destruction, while the changes of peripheral blood cells can serve as early signals of immune impairment in COVID-19 patients [26].

Glucocorticoids can avoid excessive inflammation by inhibiting immune response to SARS-Cov-2 infection, while the suppression of immunity may lead to an increase in viral load [27]. Besides, glucocorticoids can suppress the release of EOS in bone marrow and promote eosinophil clearance by directly inducing apoptosis [28, 29]. The panel of WHO made a strong recommendation for use of glucocorticoids in severe and critical COVID-19 patients, and in a real-life clinical setting, physicians tend to use glucocorticoids in most critically patients [30]. In this cohort, we proved that the use of glucocorticoids altered the immunological characteristics of peripheral blood cells and glucocorticoid-related EOS decreased, which was considered as a risk factor for fatal outcomes. As the role of glucocorticoids in treating severe COVID-19 patients is still controversial, blood immunological marker which could be used as an index to guide the strategy of glucocorticoids therapy in COVID-19 patients is needed and may improve the prognosis in the clinical practice.

Early identification of risk factors for critical illness can facilitate appropriate provision of supportive care and help reduce mortality. Blood routine seems like a convenient and effective indicator which can help to identify the entities involved in immune dysregulation. Lymphopenia and eosinopenia on admission may be particularly important to indicate the poor prognosis of COVID-19 patients, and counts of eosinophils are of guiding significance for the use of glucocorticoids. Unchanged levels of EOS during monitoring and treatment also hinted the poor prognosis of COVID-19 patients. In conclusion, peripheral white blood cells may serve as early signals of disease progression, which can be chosen as convenient and effective monitor parameters during the treatment of COVID-19.

## Supplementary Information

**Additional file 1: Table S1.** Effect of glucocorticoids on outcome of low-EOS patients. **Table S2.** Effects of EOS count on survival in groups divided by glucocorticoids use. **Table S3.** Effect of peripheral blood cell counts and change on clinical outcome.

## Data Availability

The data-sets used and/or analyzed during the current study available from the corresponding author on reasonable request.

## Abbreviations

APTT: Activated partial thromboplastin time
BASO: Basophils
CRP: C-reactive protein
COVID-19: Coronavirus disease 2019
EOS: Eosinophils
FIB: Fibrinogen
IQR: Interquartile range
LDH: Lactate dehydrogenase
LYM: Lymphocytes
MV: Mechanical ventilation
MERS-CoV: Middle east respiratory syndrome
MONO: Monocytes
NMV: Noninvasive methods of mechanical ventilation
NT-proBNP: N-terminal pro brain natriuretic peptide
NEU: Neutrophils
PT: Prothrombin time
RT-PCR: Real-time reverse transcriptase polymerase-chain reaction
SARS-CoV: Severe acute respiratory syndrome
SARS-CoV-2: Severe acute respiratory syndrome coronavirus
WBC: White blood cell
WHO: World Health Organization
