# Supplementary material for: Characteristics of peripheral white blood cells in COVID-19 patients revealed by a retrospective cohort study
Source: BMC Infect Dis. 2021 Dec 9;21:1236. doi: 10.1186/s12879-021-06899-7 (PMC8655490; doi:10.1186/s12879-021-06899-7)
Supplement: Supplementary file 1 — Additional file 1: Table S1. Effect of glucocorticoids on outcome of low-EOS patients. Table S2. Effects of EOS count on survival in groups divided by glucocorticoids use. Table S3. Effect of peripheral blood cell counts and change on clinical outcome. [file 12879_2021_6899_MOESM1_ESM.docx]

**Supplemental Table 1 Effect of glucocorticoids on outcome of low-EOS patients**

|  | Survivor  (n=65) | Non-Survivor  (n=13) | *P* value |
| --- | --- | --- | --- |
| Eosinophils(baseline), ×10^9^/L | 0.00(0.00,0.00) | 0.00(0.00,0.00) | 0.056 |
| Eosinophils(endpoint), ×10^9^/L | 0.07(0.03,0.16) | 0.00(0.00,0.00) | **<0.001** |
| Eosinophils(change), ×10^9^/L | 0.07(0.02,0.14) | 0.00(0.00,0.00) | **0.002** |
| Glucocorticoids | 22(40.0%) | 8(66.7%) | 0.117 |

**Supplemental Table 2 Effects of EOS count on survival in groups divided by glucocorticoids use.**

|  | Survivor | Non-Survivor | *P* value |
| --- | --- | --- | --- |
| **with CS use** |  |  |  |
| Numbers of patients | 34 | 8 |  |
| Eos count (baseline) , ×10^9^/L | 0.01(0.00,0.04) | 0.00(0.00,0.00) | **0.014** |
| Eos count (endpoint) , ×10^9^/L | 0.04(0.01,0.12) | 0.00(0.00,0.00) | **0.019** |
| Eosinophils(change), ×10^9^/L | 0.03(0.00,0.11) | 0.00(0.00,0.00) | 0.274 |
| **without CS use** |  |  |  |
| Numbers of patients | 117 | 4 |  |
| Eos count (baseline) , ×10^9^/L | 0.04(0.01,0.10) | 0.00(0.00,0.00) | **0.008** |
| Eos count (endpoint) , ×10^9^/L | 0.13(0.07,0.21) | 0.00(0.00,0.00) | **0.018** |
| Eosinophils(change), ×10^9^/L | 0.03(0.08,0.16) | 0.00(0.00,0.00) | **0.028** |

**Supplemental Table 3 Effect of peripheral blood cell counts and change on clinical outcome.**

|  | Survivor (n=185) | Non-survivor (n=13) | *P* value |
| --- | --- | --- | --- |
| NEU baseline, ×10^9^/L | 3.36(2.40,4.66) | 5.58(2.99,8.41) | **0.024** |
| NEU endpoint, ×10^9^/L | 3.20(2.55,4.33) | 7.16(3.64,14.98) | **0.002** |
| NEU change, ×10^9^/L | -0.13(-1.55,0.75) | 3.27(1.37,6.17) | **0.001** |
| LYM baseline, ×10^9^/L | 1.08(0.78,1.57) | 0.50(0.33,0.82) | **<0.001** |
| LYM endpoint, ×10^9^/L | 1.54(1.15,2.06) | 0.42(0.12,0.54) | **<0.001** |
| LYM change, ×10^9^/L | 0.46(0.16,0.76) | -0.14(-0.48,0.05) | **<0.001** |
| MON baseline, ×10^9^/L | 0.47(0.34,0.63) | 0.33(0.21,0.60) | 0.168 |
| MON endpoint, ×10^9^/L | 0.51(0.41,0.64) | 0.44(0.15,0.63) | 0.170 |
| MON change, ×10^9^/L | 0.01(-0.11,0.16) | -0.09(-0.20,0.13) | 0.246 |
| EOS baseline, ×10^9^/L | 0.03(0.00,0.10) | 0.00(0.00,0.00) | **<0.001** |
| EOS endpoint, ×10^9^/L | 0.11(0.06,0.20) | 0.00(0.00,0.00) | **<0.001** |
| EOS change, ×10^9^/L | 0.07(0.02,0.14) | 0.00(0.00,0.00) | **0.002** |
| BASO baseline, ×10^9^/L | 0.01(0.01,0.02) | 0.00(0.00,0.01) | **0.001** |
| BASO endpoint, ×10^9^/L | 0.02(0.01,0.04) | 0.02(0.01,0.05) | 0.811 |
| BASO change, ×10^9^/L | 0.01(0.00,0.02) | 0.02(0.01,0.04) | 0.104 |
